# *Nocardia nova* Infection of Tibia Tenodesis Implant After Anterior Cruciate Ligament Reconstruction in an Immunocompetent Patient

**DOI:** 10.5435/JAAOSGlobal-D-19-00167

**Published:** 2020-02-25

**Authors:** Porter Young, Andrew Riga, Jeffrey Brunelli

**Affiliations:** From the Department of Orthopaedic Surgery and Rehabilitation, University of Florida, Jacksonville, FL.

## Abstract

Infection after anterior cruciate ligament reconstruction and *Nocardia* infection in immunocompetent hosts are rare events in isolation. This case report highlights the rare combination of these events in a 46-year-old healthy man who acquired a *Nocardia nova* infection of the tibia tunnel site after an anterior cruciate ligament reconstruction with peroneus allograft. He was successfully treated with tibial tenodesis screw removal, two surgical debridements, and 4 weeks of trimethoprim-sulfamethoxazole and meropenem, followed by 6 months of clarithromycin and original graft retention. This report will review the current antibiotic recommendations and surgical management of this challenging situation. Our case is unique in that the infection was isolated to the distal aspect of the tibial tunnel and did not spread into the entire knee joint, highlighting the importance of early debridement and irrigation in the operative suite when graft site infection is suspected.

Anterior cruciate ligament (ACL) reconstruction with allograft tendon is a common procedure that carries a low risk of disease transmission and infection based on several key processing and handling factors. Interference screws and tenodesis implants are a possible local harbor of infection in ACL reconstruction. *Nocardia*, an opportunistic bacterium, rarely affects immunocompetent hosts. This case report highlights the rare combination of these events in an ACL reconstruction while maintaining the original allograft at the final follow-up. The *Nocardia nova* infection was successfully treated with hardware removal, antibiotics, and graft retention.

## Case Report

This case involves a 46-year-old man with a medical history of hypertension, obstructive sleep apnea, and pericarditis who sustained a left ACL tear after a noncontact twisting injury while playing basketball. After a trial of physical therapy and anti-inflammatory medication, he still experienced persistent pain and instability and elected to undergo arthroscopic ACL reconstruction with a peroneus allograft. The graft was fixed to the femur using a suture button and the tibial side was fixed with a tenodesis implant. No complications were noted intraoperatively.

His immediate postoperative course was uneventful. He experienced intermittent knee swelling that was activity related, but this was not associated with inability to bear weight or other signs concerning for infection. However, 4.5 months postoperatively, he developed acute anterior tibial swelling with associated warmth and erythema at the tibial tunnel site. This was lanced at an outside facility after they suspected it to be an abscess, and he was placed on oral clindamycin and trimethoprim-sulfamethoxazole (TMP-SMX). He presented to our clinic 5 days later with persistent drainage from the wound and exposed tenodesis implant in the wound.

He then underwent formal debridement and irrigation with tenodesis implant removal and primary wound closure in the operating room. Gram stain from intraoperative specimens demonstrated few neutrophils, but no organisms. Infectious disease was consulted, and empiric intravenous vancomycin and piperacillin/tazobactam were started. However, by postoperative day 4, cultures grew *N nova*. Empiric antibiotics were stopped, and he was transitioned to oral TMP-SMX and intravenous meropenem.

He was doing well until 4 weeks after the debridement when he developed erythema, drainage, and wound dehiscence. He also developed an acute renal injury, so sulfamethoxazole-trimethoprim was switched to minocycline per infectious disease recommendations. He underwent a repeat debridement where a brownish fluid collection had developed around a braided suture tied to the end of the ACL graft. The suture was excised and wound thoroughly debrided. A diagnostic arthroscopy was then performed to rule out an intra-articular source of infection. It demonstrated an intact ACL graft and no evidence of the infection spreading from the tibial tunnel into the joint. Cultures taken at this debridement showed no growth, and he was transitioned to oral clarithromycin alone for 6 months. His wound healed without issues. His knee pain resolved, and he was able to return to his previous level of activity playing recreational basketball.

## Discussion

*Nocardia* belongs to the family of aerobic actinomycetes and is an acid-fast positive, branching filamentous bacillus. It is commonly found in water, soil, and in decaying organic matter.^1^ The incidence of infection in the United States is estimated to be 500 to 1,000 cases per year and is more common in immunocompromised hosts. The most common routes of infection are through skin or the respiratory tract, where it disseminates to other organ systems. Mortality ranges from 20% in the immunocompetent host and up to 55% in immunocompromised hosts. *Nocardia* is a slowly growing bacterium that can take 7 to 30 days to grow on culture media, thus highlighting the importance of informing the microbiology laboratories to grow samples longer than the standard 5 days. However, DNA sequencing is required to identify the specific species of *Nocardia* and to determine antibiotic susceptibilities.^2^

TMP-SMX has been considered the historical standard antimicrobial therapy for *Nocardia* species with multiple studies reporting only a 2% to 3% resistance.^3-5^ However, the Center for Disease Control published susceptibilities of 765 isolates of *Nocardia* and found that 53% of *N nova* isolates were resistant to TMP-SMX.^6^ For severe infections or in immunocompromised patients, combination therapy with TMP-SMX plus either an aminoglycoside, third-generation cephalosporin, minocycline, clarithromycin or carbapenem is recommended based on susceptibility patterns.^7^ Although meropenem is effective against some isolates of *Nocardia*, *N nova* has demonstrated increasing resistance to it. This may explain why our patient's symptoms recurred after 4 weeks of TMP-SMX and meropenem.^6,7^ *N nova* resistance to minocycline has been reported as high as 54%.^6^ The final antibiotic that helped eradicate our patient's infection, clarithromycin, has only demonstrated a 4% to 7% rate of resistance to *N nova*.^6,8^ Given the relapsing nature of *Nocardia*, most infectious disease experts recommend antibiotics for 6 to 12 months depending on the severity of the disease.^5^ The numerous antibiotic options and their various efficacies against *Nocardia* highlight the importance of including input from an infectious disease consultant and obtaining formal antimicrobial susceptibilities for all *Nocardia* infections.

Infections after ACL reconstruction are rare, occurring in approximately 1.0% of cases, but they can be difficult to treat and eradicate. *Staphylococcus aureus* is the most common cause occurring in 65% of infected cases.^9,10^ Allografts carry a theoretical higher risk of infection compared with autografts due to the fact that a foreign object is introduced into the knee. However, Katz et al^11^ performed a retrospective review of 801 patients to compare the infection rate between ACL reconstruction with autograft versus allograft. Their incidence of infection was 1.2% with autografts versus 0.6% with allografts which is contrary to the common belief that allografts carry a higher infection risk than autografts. Multiple other studies have reached a similar conclusion.^10,12^

There have been only two reported cases of ACL reconstructions becoming infected with *Nocardia*, both of which used allograft tendons.^13,14^ Gupta and Moorman^13^ reported the first case in 2010. It involved an immunocompetent 27-year-old man who developed a *N nova* infection 3 months after ACL reconstruction with a tibialis anterior allograft. He was treated with arthroscopic irrigation and debridement and the removal of all implants and the graft followed by 7 months of oral clarithromycin. Once he was determined to be infection free, he underwent an uncomplicated ACL reconstruction revision with a bone-patellar tendon-bone autograft.

In 2015, Yong et al^14^ reported an ACL reconstruction revision in a healthy 27-year-old man complicated by *Nocardia aobensis* infection. He did well until 2 months postoperatively when he developed erythema, pain, and an abscess about his surgical scar. Attempts were made to save the graft with operative debridement alone, but this failed and all hardware and allograft were eventually removed. He completed 12 months of oral TMP-SMX. Cultures from the repeat debridements were negative, and the patient was able to make a full recovery returning to his previous level of activity.

These two cases share important similarities with our case. First, late knee swelling after ACL reconstruction should always raise the concern for infection because indolent organisms such as *Nocardia* can take weeks to present. Second, surgeons should counsel patients that eradication of *Nocardia* requires patience because it can take multiple debridements and months of antibiotics to eliminate the infection. However, our case is novel in that it is the first reported case of a *Nocardia* infection after ACL reconstruction successfully treated with aggressive surgical debridement, graft retention, and long-term antibiotics. In fact, recent literature suggests that patients may have better outcomes when grafts can be retained in the setting of an infected ACL reconstruction.^15^

The source of our patient's infection could be multifactorial, and we will never know the true source. Graft contamination is always a concern in reconstructions with allografts. However, the infection was isolated to the distal aspect of his tibial tunnel and did not spread into the knee joint making this an unlikely source. The tenodesis screw could have been contaminated at some point during the procedure but this is also unlikely. The authors suspect that our patient likely contracted the infection during or after the debridement of his tibial wound by the outside facility. Given that *Nocardia* is a common environmental bacterium, it likely contaminated his open wound before arriving at our facility. Regardless of the source, our case is unique in that the infection was isolated to the distal aspect of the tibial tunnel and did not spread into the entire knee joint highlighting the importance of early debridement and irrigation in the operative suite when graft site infection is suspected.
